# Tomato Plant Microbiota under Conventional and Organic Fertilization Regimes in a Soilless Culture System

**DOI:** 10.3390/microorganisms11071633

**Published:** 2023-06-22

**Authors:** Carolina N. Resendiz-Nava, Fernando Alonso-Onofre, Hilda V. Silva-Rojas, Angel Rebollar-Alviter, Dulce M. Rivera-Pastrana, Matthew J. Stasiewicz, Gerardo M. Nava, Edmundo M. Mercado-Silva

**Affiliations:** 1 Facultad de Quimica, Universidad Autonoma de Queretaro, Cerro de las Campanas S/N, Queretaro 76010, Queretaro, Mexico; 2 Centro Universitario CEICKOR, Colon 76299, Queretaro, Mexico; 3 Posgrado en Recursos Geneticos y Productividad, Produccion de Semillas, Colegio de Postgraduados, Km 36.5 Carretera Mexico-Texcoco, Texcoco 56264, Mexico; 4 Centro Regional Morelia, Universidad Autonoma de Chapingo, Morelia 58170, Michoacan, Mexico; 5 Department of Food Science and Human Nutrition, University of Illinois at Urbana-Champaign, 1302W Pennsylvania Ave, Urbana, IL 61801, USA

**Keywords:** *16S rRNA*, core microbiota, organic, phyllosphere, rhizosphere, soilless, tomato

## Abstract

Tomato is the main vegetable cultivated under soilless culture systems (SCSs); production of organic tomato under SCSs has increased due to consumer demands for healthier and environmentally friendly vegetables. However, organic tomato production under SCSs has been associated with low crop performance and fruit quality defects. These agricultural deficiencies could be linked to alterations in tomato plant microbiota; nonetheless, this issue has not been sufficiently addressed. Thus, the main goal of the present study was to characterize the rhizosphere and phyllosphere of tomato plants cultivated under conventional and organic SCSs. To accomplish this goal, tomato plants grown in commercial greenhouses under conventional or organic SCSs were tested at 8, 26, and 44 weeks after seedling transplantation. Substrate (*n* = 24), root (*n =* 24), and fruit (*n =* 24) composite samples were subjected to DNA extraction and high-throughput *16S rRNA* gene sequencing. The present study revealed that the tomato core microbiota was predominantly constituted by Proteobacteria, Actinobacteria, and Firmicutes. Remarkably, six bacterial families, *Bacillaceae*, *Microbacteriaceae*, *Nocardioidaceae*, *Pseudomonadaceae*, *Rhodobacteraceae*, and *Sphingomonadaceae*, were shared among all substrate, rhizosphere, and fruit samples. Importantly, it was shown that plants under organic SCSs undergo a dysbiosis characterized by significant changes in the relative abundance of *Bradyrhizobiaceae*, *Caulobacteraceae*, *Chitinophagaceae*, *Enterobacteriaceae*, *Erythrobacteraceae*, *Flavobacteriaceae*, *Nocardioidaceae*, *Rhodobacteraceae*, and *Streptomycetaceae*. These results suggest that microbial alterations in substrates, roots, and fruits could be potential factors in contributing to the crop performance and fruit quality deficiencies observed in organic SCSs.

## 1. Introduction

Tomato (*Solanum lycopersicum* L.) fruit is one of the most consumed vegetables around the world, with an estimated production of ∼160 million tons per year [1,2,3]. In recent years, production of tomatoes under soilless culture systems (SCSs), hosted in greenhouses, has emerged as a sustainable and intensive agricultural practice [4,5]. It has been estimated that ∼95% of tomatoes produced in greenhouses from Europe and North America are cultivated under SCSs [5].

In SCSs, soil is replaced by inert substrates (e.g., coconut fiber, peat, perlite, rockwool, and struvite) and nutrients are supplied through irrigation water [4,6]. Tomato production under SCSs provides numerous advantages, for example, efficient use of water and nutrients, increased fruit quality, off season production, reduction of soil-borne pathogens, and low environmental impact [4,5,7]. Importantly, it has been estimated that SCSs generate 30% higher fruit yields than soil-based cultivation systems [4]. Additionally, SCSs require 75% less water (∼50 L/kg of tomato fruits) than soil-based production (∼200 L/kg of tomato fruits) [5]; for these reasons, the SCS is considered a more sustainable intensive production system [4,6].

In the last decade, production of organic tomato fruits has increased considerably due to consumer demands for healthier and safer vegetables [8]. Because certified organic farming strictly prohibits the use of synthetic fertilizers, fungicides, herbicides, and pesticides [9], organic tomatoes are considered as a healthier alternative [10].

Unfortunately, it has been documented that tomato plants under organic farming undergo moderate environmental stress [11], causing lower fruit yields [12,13,14,15,16], a reduction in fruit weight and firmness [14,16], and an increase in phytopathogen infections [9,17], compared to conventional production systems. Interestingly, it has been suggested that a potential cause of the agricultural problems observed in organic production could be associated with alterations of the tomato plant microbiota [18,19,20].

An imbalance in microbial populations (dysbiosis) in roots (rhizosphere) and aerial plant surfaces (phyllosphere) could promote plant disease [21,22,23] and negatively impact tomato plant growth and productivity [19,24]; thus, it could be possible that tomato plants under SCSs experience alterations to their core microbial populations. A core microbiota is defined as microbial taxa that remains stable independently of plant genotype and environmental conditions [23]. Based on this premise, the main goal of the present study was to characterize the rhizosphere and phyllosphere microbiota of tomato plants cultivated under conventional and organic fertilization regimes in SCSs and to gain insights into the potential impact of these agricultural practices on the establishment of potentially beneficial and pathogenic bacterial populations.

## 2. Materials and Methods

### 2.1. Tomato Plants Grown in Soilless Culture Systems

The study was conducted in two commercial greenhouses located in Queretaro, Mexico (20°42′22.5″ N 99°56′27.6″ W) during the 2019 growing season. Seeds of *Solanum lycopersicum* cv. “Merlice” were grown in commercial rockwool substrates; 16 days after germination, seedlings were grafted on *Solanum lycopersicum* cv. “Maxifort” (DeRuiter, The Netherlands). Two weeks after grafting, two seedlings were transplanted into a commercial soilless grow bag. Grow bags had a dimension of 110 cm × 20 cm × 12 cm and contained growing substrates made of sterile coconut fiber (Galaku International, Vaucluse, NSW, Australia); substrates had a 40% air filled porosity, 55% water holding capacity, 40% water retention efficiency, and a 30 mL 10 min^−1^ capillary uptake value.

For the conventional and organic SCSs, a drop irrigation system was used to provide plants with nutrients; for the conventional system, nutrient solution contained Ca(NO₃)₂ (123 g L^−1^), CaCl_2_ (9 g L^−1^), Ca-EDTA (2 g L^−1^), KNO_3_ (26 g L^−1^), KCl (5 g L^−1^), K_2_SO_4_ (33 g L^−1^), MgSO_4_ (48 g L^−1^), KH_2_PO_4_ (21 g L^−1^), and a mixture of the commercial fertilizers Quelsel Mix (6.5% Fe, 2.1% Mg, 0.4% Zn, 0.2% Cu, 2% B, 0.1% Mo, 88.7% chelating agents) (3.5 g L^−1^), Newquel Mn 13% (0.14 g L^−1^), Newquel Zn 14% (0.405 g L^−1^), and Quelsel Fe 6% (1 g L^−1^) (Diosol, Mexico) [1,19].

For the organic SCS, the nutrient solution contained CaSO_4_ (50 g L^−1^), MgSO_4_ (66 g L^−1^), and K_2_SO_4_ (90 g L^−1^); additionally, an organic fertilizer, Tierra Fertil 5-7-1 (55 mL L^−1^, Mar y Tierra, Mexico) was added [1,19]. All organic nutrients were obtained from providers registered in the Organic Material Review Institute (OMRI), and production management was in accordance with the National Organic Program (NOP) from the United States Department of Agriculture (USDA) [25].

Nutrient solutions for the conventional and organic SCSs were adjusted to maintain the concentration of the following nutrients: Ca (7.3 mM), Cl (9 mM), K (7 mM), Mg (2.8 mM), NH_4_ (0.8 mM), NO_3_ (12.5 mM), PO_4_ (2 mM), and SO_4_^−2^ (3.4 mM); as well as the microelements: B (0.09 mM), Cu (0.001 mM), Fe (0.044 mM), Mn (0.013 mM), Mo (0.001 mM), and Zn (0.009 mM) [1,19]. Additionally, the following commercial pathogen control agents were supplemented in conventional and organic SCSs AgroClean (Koppert, Berkel en Rodenrijs, The Netherlands), Amifol K (Tradecorp, Zapopan, Mexico), Kumulus (BASF, Mexico City, Mexico), MilStop (PHC, Mexico City, Mexico), Serenade (Bayer CropScience, Leverkusen, Germany), and System Cu (Idai Nature, Valencia, Spain) as indicated by the manufacturers. The electrical conductivity and pH of the nutrient solution in the conventional fertilization regime were, on average, 2.6 mS cm^−1^ and 6.2, respectively; whereas, in the organic fertilization regime, they were 1.84 mS cm^−1^ and 6.6, respectively. Average day/night temperature and relative humidity inside greenhouses were ~23/17 °C, and ~86/92%, respectively. The tomato fruit production cycle comprised weeks 8 to 44, after seedlings transplantation (AST). During this period, twelve plants from each production system were randomly selected and labeled across the whole greenhouse. Substrate, rhizosphere, and tomato fruit samples from these selected plants were collected for microbial analyses. Overall, tomato yield under organic SCSs was, on average, 19% lower than the conventional fertilization regime (unpublished data).

### 2.2. Sample Collection and DNA Extraction

Substrate (*n =* 12), rhizosphere (*n =* 12), and fruit (*n =* 12) samples from plants grown in each SCS, conventional and organic, were collected at weeks 8, 26, and 44 AST (Table 1). Substrate samples (~5 g) were collected from root-free zones (>1 cm from roots) as previously described [18,19]. Rhizosphere samples (~2 g) were collected ~10 cm away from the stem; roots were shaken to remove loose substrate particles, and only bacterial communities associated within ~1 mm of the root surface remained [18,26,27]. A tomato sample was composed of three fruits collected from a single cluster harvested at the pink maturity stage and grade 4, according to the USDA color classification requirements [28]. All samples were collected aseptically, using sterile gloves and sampling bags; placed in a cooler (~4 °C); and transported to the laboratory within 3 h of collection.

To gain insights into the core microbiota, the thirty-six substrate, rhizosphere, and fruit samples collected during the whole production cycle (from weeks 8 to 44 AST) were processed as composite samples (3 individual samples = 1 composite sample) (Table 1). Composite samples (*n* = 12) were subjected to DNA extraction; briefly, the substrate (1.0 g), rhizosphere (1.0 g), and tomato samples were rinsed with sterile deionized water (1.0, 1.0, and 3.0 mL, respectively) for ~1 min to collect microbial populations associated with the samples [26,29,30]. Sample washes were centrifuged at 8000× *g* during 1 min and the obtained pellets (~200 mg) were subjected to total DNA extraction using the ZymoBIOMICS^®^ DNA Miniprep kit, (Zymo Research, Irvine, CA, USA) following the manufacturer’s instructions. DNA concentration and quality were measured using a Nanodrop 2000 UV-Vis spectrophotometer (Thermo ScientificTM, Waltham, MA, USA). All DNA samples were diluted at 5 ng/μL and stored at −20 °C.

### 2.3. High-Throughput Sequencing of 16S rRNA and Data Processing

A total of 72 samples were subjected to next-generation sequencing (NGS) using the ZymoBIOMICS^®^ Targeted Sequencing Service for Microbiome Analysis at the Zymo Research company (https://zymoresearch.eu/pages/16s-its-amplicon-sequencing; Irvine, CA, USA; accessed on 1 April 2023) [31,32,33,34]; briefly, V3–V4 regions of the *16S rRNA* gene were PCR amplified and then used for sequence library construction using the *Quick*-16S™ NGS Library Prep Kit (Zymo Research, Irvine, CA, USA), as described elsewhere [35]. Sequencing via synthesis was performed with the Illumina^®^ MiSeq™ platform (San Diego, CA, USA) [36]. After sequencing, primers and adaptor sequences were removed from the reads, and the sequences were trimmed to the same length (~320 bases, using Illumina Data Analysis Software V2.3) [37]. Low-quality reads and chimeric sequences were removed using the Divisive Amplicon Denoising Algorithm 2 (DADA2) pipeline [38]. Rarefaction curves were generated based on the number of bacterial Amplicon Sequence Variants (ASVs) to confirm that sampling depth was sufficient and that all samples reached a plateau. ASVs were classified at the phylum and family level using UCLUST from QIIME v.1.9.1 [39] using the Zymo Research Database, an internally designed and curated reference database [31,32,33,34,35].

### 2.4. Analyses of Core Microbiota, Bacterial Abundance, and Diversity

Analyses of core microbiota at each selected niche were performed using relative abundance and occurrence data with cut-off values of ≥1% and ≥50%, respectively, as previously described [40]. The relative abundance of the total bacteria was estimated by means of the *16S rRNA* gene copy number generated for each sample using quantitative PCR (qPCR) assays with the absolute quantification method; for this goal, a *16S rRNA* gene plasmid-DNA standard curve was used for the analysis. The total gene copy number was calculated using the following equation: number of copies = [amount of DNA (ng) × Avogadro’s number (6.022 × 10^23^)]/[estimated genome size (4.64 × 10^6^ bp*) × average molecular weight of a DNA bp (660 g/mole/bp)] [41,42,43,44].

Analyses of alpha diversity, richness, Simpson, and evenness indices [45,46,47] were estimated using bacterial relative abundance data at the family level. Beta diversity was estimated using Bray–Curtis and Jaccard indices [48,49]. Comparisons of microbiota composition were carried out using Principal Component Analysis (PCA) [50].

Identification of potential bacterial biomarkers was performed by using linear discriminant analysis (LDA) effect size (LEfSe), an approach highlighting biological consistency and effect size relevance [51]. LDA-LEfSe was performed using the all-against-all mode, calculating the LDA score after 200 bootstrapping iterations and a level of significance ≤ 0.05. The LDA score threshold was set to 4.0 [51,52,53].

### 2.5. Absolute Quantification of Enterobacteriaceae in Tomato Fruits

An independent set of tomato fruit samples were collected from a different harvest season, six months after the initial experiment. DNA from tomato fruits was extracted as described above; this DNA was subjected to quantification of *Enterobacteriaceae* by means of quantitative PCR (qPCR) analysis using primers PS1-forward (5′-GGGGATAACYACTGGAAACGGTRGC-3′) and PS1-reverse (5′-GCATGGCTGCATCA GGSTTKC-3′); these primers amplified a ~236 bp segment of the *16S rRNA* gene, previously validated [54,55]. Each qPCR reaction included 5 μL of Takara SYBR^®^ Premix Ex Taq™ (Takara Bio Inc., Shiga, Japan), 0.68 μL of each primer (1.0 μM), 0.85 μL of bovine serum albumin (Bioline, London, UK), 10.0 ng of sample DNA, and nuclease-free water for a total volume of 17 μL. The amplification program consisted of 1 min at 95 °C followed by 35 cycles of denaturing at 95 °C for 30 s, annealing at 60 °C for 30 s, an extension at 72 °C for 30 s., and a final extension for 2 min at 72 °C. A qPCR standard curve was generated using DNA extracted from a *E. coli* reference strain (ATCC 11229).

### 2.6. Statistical Analyses

Results were also analyzed by means of an unpaired *t*-test and ANOVA Fisher’s protected least significant difference test using StatView version 5.0.1. Differences were considered significant at *p* ≤ 0.05. Alpha and beta diversity indices as well as PCA analyses were performed with the PAST software (v. 4.09) [56]. 

## 3. Results

### 3.1. Tomato Core Microbiota

**Figure 1:**
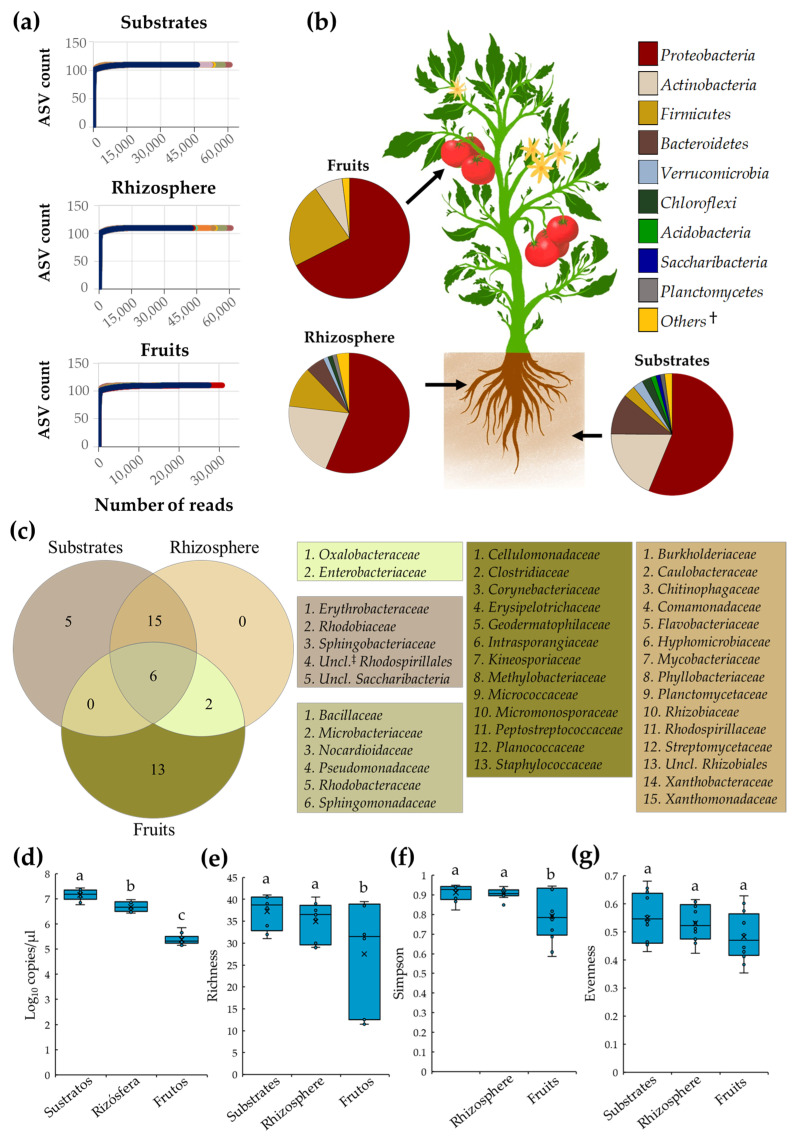
Diversity and core microbiota of tomato plants cultivated under SCSs. (**a**) Rarefaction curves for observed ASVs; (**b**) diversity of core microbiota at the phylum level; (**c**) Venn diagram depicting bacterial families shared between substrate, root, and fruit samples; boxplots of (**d**) total bacteria, (**e**) richness, (**f**) Simpson, and (**g**) evenness indices in substrates, rhizosphere, and fruit samples. Boxes indicate the interquartile range of the data; the solid line and the cross inside the box depict the median and average; respectively. Whiskers represent minimum and maximum values; circles within or outside boxes show data distribution. Different letters indicate significant differences (*p* ≤ 0.05) between samples (*n* = 24). †: Bacterial groups with relative abundance < 1%. ‡: Unclassified.

In the present study, tomato plant microbiota grown with conventional and organic SCSs was characterized after three independent samplings, during a whole production cycle. A total of 1,984,561 high-quality reads were derived from the 72 composite samples collected; from these, 613,355, 635,779, and 735,427 reads were obtained from substrate, rhizosphere, and fruit samples, respectively. Rarefaction analyses revealed that sequencing reached a plateau with the number of reads obtained from each niche, suggesting a high coverage of the tomato microbiota (Figure 1a). Microbiota of substrate, rhizosphere, and fruit samples was composed by nine, seven, and three major bacterial phyla (listed in Figure 1b), respectively, from which Proteobacteria was the most abundant microbial member in substrate (56.2%), rhizosphere (56.3%), and tomato fruit samples (67.5%) (Figure 1b).

Analysis of core microbiota revealed that substrate, rhizosphere, and tomato fruit niches were inhabited by 26, 23, and 21 bacterial families, respectively. Interestingly, six families, *Bacillaceae*, *Microbacteriaceae*, *Nocardioidaceae*, *Pseudomonadaceae*, *Rhodobacteraceae*, and *Sphingomonadaceae*, were shared among all selected niches. As expected, substrates and roots shared the largest number of families (15 taxa), suggesting a close biological interaction between these two niches. In contrast, roots and fruits only shared two families, *Enterobacteriaceae* and *Oxalobacteraceae*. Additionally, it was revealed that substrates and fruits were colonized by five and thirteen bacterial families, respectively. Notably, it was found that rhizosphere possesses no unique bacterial families; all members of root-associated microbiota were shared with substrates and fruits (Figure 1c).

Analyses of tomato plant bacterial diversity revealed that substrates had the highest (*p* ≤ 0.05) relative abundance of total bacteria (average 7.2 log_10_), measured by *16S rRNA* gene copies, followed by rhizosphere (average 6.7 log_10_), and fruit (average 5.4 log_10_) samples (Figure 1d). Interestingly, the bacterial diversity between substrate and root samples was comparable (*p* > 0.05) as indicated by richness, Simpson, and evenness indices, corroborating a close biological interaction between these two niches. In contrast, bacterial diversity indices in tomato fruit samples were lower (*p* ≤ 0.05) compared to substrate and root samples, suggesting that this niche was colonized by a lessened and uneven number of bacterial families (Figure 1d–g).

### 3.2. Differences in Tomato Microbiota between Conventional and Organic Soilless Culture Systems

**Figure 2:**
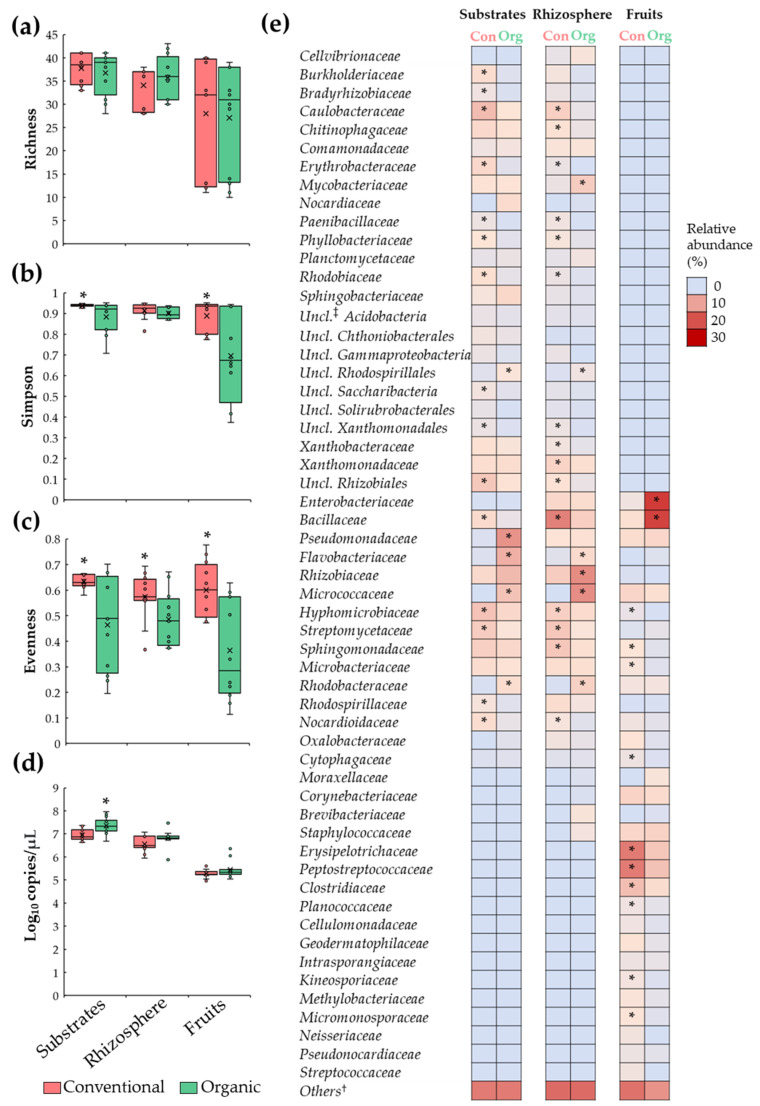
Comparisons of tomato plant microbiota between conventional (Con) and organic (Org) fertilization regimes in SCSs. Boxplots depicting (**a**) richness, (**b**) Simpson, (**c**) and evenness indices as well as (**d**) total bacteria. Features of the boxplots are described in Figure 1. (**e**) Heatmap portraying relative abundance of bacterial families in substrate, rhizosphere, and tomato fruit samples. Asterisks indicate significant differences (*p* ≤ 0.05) between samples (*n =* 12). †: Bacterial groups with relative abundance < 1%. ‡: Unclassified.

Analyses of microbiota diversity revealed a comparable (*p* > 0.05) number of bacterial families (richness) in substrate, rhizosphere, and fruit samples between conventional and organic SCS; also, it was observed that the family richness index was more variable among fruit samples (Figure 2a). Moreover, it was revealed that substrates and fruit samples under organic SCSs had a lower Simpson diversity index compared to their conventional counterparts (Figure 2b), suggesting that bacterial populations, in organic samples, are unevenly distributed. This observation was corroborated via evenness index analysis, showing a reduction (*p* ≤ 0.05) in this parameter in organic samples when compared to conventional samples (Figure 2c). Importantly, analysis of the relative abundance revealed minor (<1 log_10_) or no differences (*p* > 0.05) in total bacterial density, measured by *16S rRNA* gene copies, between organic and conventional samples (Figure 2d). Together, these data indicate that substrates, rhizosphere, and fruit samples from organic and conventional SCSs are colonized by a comparable number of microbial taxa; however, in organic samples, a few bacterial groups are predominantly abundant.

To corroborate the difference in the relative abundance of bacterial families between conventional and organic systems, inferential and multivariate statistics were performed. These analyses revealed that substrates from the organic system were enriched (*p* ≤ 0.05) by members of *Flavobacteriaceae*, *Micrococcaceae*, *Pseudomonadaceae*, *Rhodobacteraceae*, and unclassified *Rhodospirillales* families; and reduced (*p* ≤ 0.05) in members of *Bacillaceae*, *Burkholderiaceae*, *Bradyrhizobiaceae*, *Caulobacteraceae*, *Erythrobacteraceae*, *Hyphomicrobiaceae*, *Nocardioidaceae*, *Paenibacillaceae*, *Phyllobacteriaceae*, *Rhodobiaceae*, *Rhodospirillaceae*, *Streptomycetaceae*, unclassified *Rhizobiales*, unclassified *Saccharibacteria*, and unclassified *Xanthomonadales* compared to conventional samples. Additionally, rhizosphere samples from the organic system were enriched (*p* ≤ 0.05) by members of *Flavobacteriaceae*, *Micrococcaceae*, *Mycobacteriaceae*, *Rhizobiaceae*, *Rhodobacteraceae,* and unclassified *Rhodospirillales*; and reduced in *Bacillaceae*, *Caulobacteraceae*, *Chitinophagaceae*, *Erythrobacteraceae*, *Hyphomicrobiaceae*, *Nocardioidaceae*, *Paenibacillaceae*, *Phyllobacteriaceae*, *Rhodobiaceae*, *Sphingomonadaceae*, *Streptomycetaceae*, unclassified *Rhizobiales*, unclassified *Xanthomonadales*, *Xanthobacteraceae*, and *Xanthomonadaceae* when compared to conventional samples. Moreover, it was shown that tomato fruit samples from organic SCS were enriched with (*p* ≤ 0.05) *Bacillaceae* and *Enterobacteriaceae*; and reduced in *Clostridiaceae*, *Cytophagaceae*, *Erysipelotrichaceae*, *Hyphomicrobiaceae*, *Kineosporiaceae*, *Microbacteriaceae*, *Micromonosporaceae*, *Peptostreptococcaceae*, *Planococcaceae*, and *Sphingomonadaceae* compared with conventional samples (Figure 2e). Together, these results suggest that the organic fertilization regime induced dysbiosis in substrate, rhizosphere, and fruits microbial populations.

**Figure 3:**
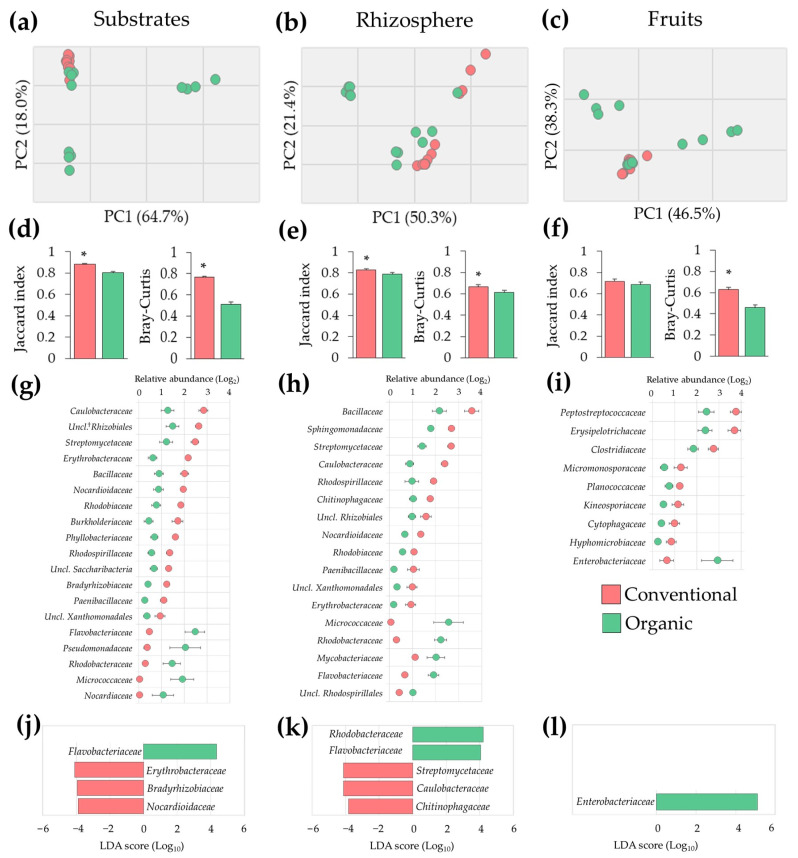
Tomato plant microbiota alterations in organic compared to conventional fertilization regimes in SCSs. (**a**–**c**) Principal Component Analysis (PCA) of microbial populations; (**d**–**f**) beta diversity indices; (**g**–**i**) altered microbial populations (1.5-fold change and *p* ≤ 0.05); and (**j**–**l**) microbial markers identified via LDA-LEfSe analysis of substrate, rhizosphere, and fruit samples. Asterisks indicate significant differences (*p* ≤ 0.05) between samples (*n =* 12). †: Unclassified.

The unbalance in microbial populations observed in samples from organic SCS was corroborated via multivariate analysis. PCA of microbiota profiles revealed a higher variability in substrate, rhizosphere, and fruit samples from the organic system when compared to conventional samples (Figure 3a–c). These results were confirmed via analyses of beta diversity, where samples from the organic system had lower (*p* ≤ 0.05) Jaccard and Bray–Curtis similarity indices when compared to conventional samples, indicating a higher variability in prevalence and relative abundance of microbial populations (Figure 3d–f). Taken together these results suggest that plants cultivated under organic SCS exhibited microbial dysbiosis in roots and fruits.

### 3.3. Microbial Dysbiosis Biomarkers

To gain insights into the nature of the dysbiosis observed in the organic SCS, bacterial densities between conventional and organic samples were compared. These analyses revealed, at least 1.5-fold change (FC) differences (*p* ≤ 0.05) in nineteen, seventeen, and nine families in substrate, rhizosphere, and fruit samples, respectively. Many of them exhibiting inversely differential abundance; in substrates, the most drastic changes were observed in *Micrococcaceae* (63.1 FC) and *Nocardiaceae* (37.1 FC), followed by *Pseudomonadaceae* (5.7 FC), *Flavobacteriaceae* (5.3 FC), and *Rhodobacteraceae* (5.3 FC). In the rhizosphere, major differences were observed in *Micrococcaceae* (48.6 FC), followed by *Rhodobacteraceae* (7.5 FC), *Paenibacillaceae* (5.3 FC), and *Erythrobacteraceae* (5.3 FC). In fruits, significant differences were observed in *Enterobacteriaceae* (4.6 FC) and *Hyphomicrobiaceae* (4 FC) (Figure 3g–i). 

Because these microbial communities showed remarkably higher beta diversity variability, analyses of LDA-LEfSe were carried out to identify potential biomarkers explaining most of the microbial effects between conventional and organic SCSs. This approach revealed that four, five, and one families were differentially (*p* ≤ 0.05) abundant with a LDA score of 4.0 in substrate, rhizosphere, and fruit samples, respectively. Specifically, a significant (*p* ≤ 0.05) enrichment of *Flavobacteriaceae* and a marked (*p* ≤ 0.05) reduction of *Erythrobacteraceae*, *Bradyrhizobiaceae*, and *Nocardioidaceae* were observed in organic substrate samples. Additionally, an increased (*p* ≤ 0.05) abundance of *Rhodobacteraceae* and *Flavobacteriaceae* and a reduction (*p* ≤ 0.05) of *Streptomycetaceae*, *Caulobacteraceae*, and *Chitinophagaceae* were observed in organic rhizosphere samples. Moreover, a marked (*p* ≤ 0.05) enrichment of *Enterobacteriaceae* in organic tomato fruits was observed (Figure 3j–l). Taken together these results indicate that plants under the organic SCS undergo a dysbiosis process in roots and fruits. Importantly, some of these changes in bacterial densities could be used as potential biomarkers to evaluate the health and performance of tomato production systems.

### 3.4. Absolute Quantification of Enterobacteriaceae in Tomato Fruits

**Figure 4:**
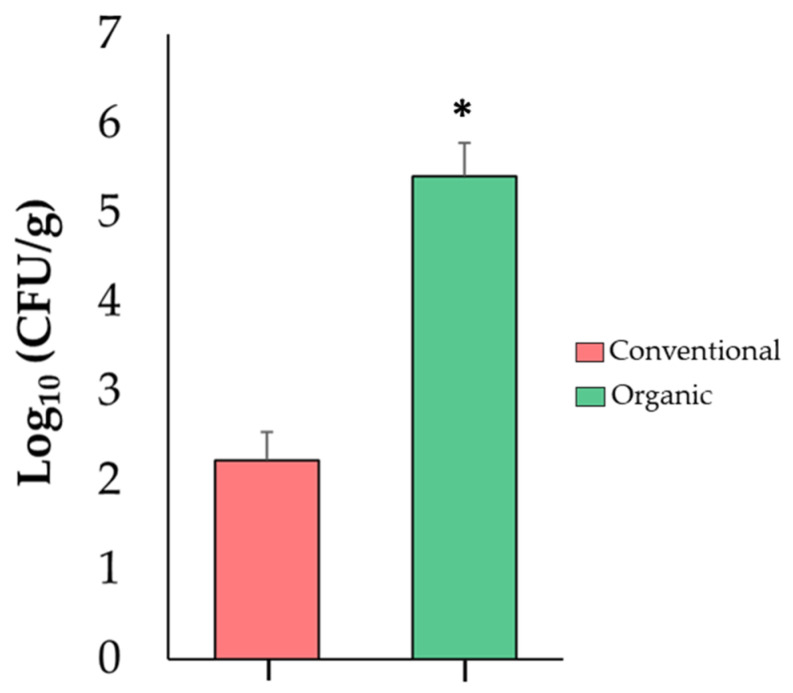
Abundance of *Enterobacteriaceae* in tomato fruit samples from conventional and organic SCSs. Asterisk indicates significant differences (*p* ≤ 0.05) between samples (*n =* 4).

Because the increase in the relative abundance of *Enterobacteriaceae* in tomato fruits is highly important for food safety, this finding was confirmed via an independent analysis using a qPCR assay. This analysis revealed that organic tomatoes were colonized by higher numbers (*p* ≤ 0.05) (5.41 log_10_) of *Enterobacteriaceae* when compared to conventional fruits (2.23 log_10_) (Figure 4). These results suggest that an organic SCS favors proliferation of *Enterobacteriaceae* in tomato fruits and could have an impact on fruit quality and shelf life. 

## 4. Discussion

Assembly of core microbiota in crop plants is driven by biotic and abiotic factors, including climate conditions, growth stage, and different fertilization regimes [57,58,59]. Numerous studies have shown that microbial populations in soil, the rhizosphere, and the phyllosphere impact crop health, productivity, and safety [60,61,62]; however, there is limited information regarding diversity of microbial populations in SCSs. This topic is of particular importance due to the advantages and opportunities provided by soilless agriculture [5]. Thus, herein we identified the core microbiota associated with tomato plants cultivated under SCSs and microbial differences between conventional and organic fertilization regimes. 

### 4.1. Tomato Core Microbiota in Soilless Culture Systems

Our data revealed that tomato core microbiota was dominated by phyla Proteobacteria, Actinobacteria, and Firmicutes in the substrate, rhizosphere, and fruit samples; comparable bacterial diversity has been reported in numerous studies with tomato plants cultivated in soil-based and SCSs [20,27,63,64,65,66,67,68,69,70]. Together, these findings suggest that, regardless of the cultivation system, tomato plants have evolved a close biological interaction with members of Proteobacteria, Actinobacteria, and Firmicutes. Bacterial groups within Proteobacteria possess a high genomic plasticity, facilitating fast growth and stress adaptation; due to these two features, Proteobacteria successfully colonize plant niches [71]. Furthermore, it has been shown that Proteobacteria harbor genomic traits linked to multiple bacteria–host beneficial processes, such as nitrogen fixation and phosphate solubilization promoted by nitrogenase and pyrroloquinoline quinone-encoding gene expression, respectively [72]. On the other hand, members of Actinobacteria and Firmicutes possess genetic features allowing for production and secretion of bacterial metabolites such as antibiotics, phytohormones, and siderophores capable of promoting plant growth [73,74,75,76]. Collectively, these bacterial groups produce metabolites that induce plant growth and disease resistance [21,72,76,77]. Taken together, these results corroborate that Proteobacteria, Actinobacteria, and Firmicutes are main components of the tomato plant’s core microbiota.

Importantly, the present study revealed that the core microbiota, during the whole production cycle, were integrated by 26, 23, and 21 families (listed in Figure 1c) in the substrate, rhizosphere, and tomato fruits, respectively. The presence of these bacterial families in tomato plants has been observed in other studies [19,26,30,66]. Interestingly, six bacterial families, *Bacillaceae*, *Microbacteriaceae*, *Nocardioidaceae*, *Pseudomonadaceae*, *Rhodobacteraceae*, and *Sphingomonadaceae*, colonized the substrate, rhizosphere, and fruit samples, suggesting the presence of a tomato plant core microbiota. Members of this core microbiota perform important functions linked to plant growth promotion; for example, *Bacillaceae* and *Pseudomonadaceae* produce metabolites such as polyketides and pyoverdines, which reduce pathogen colonization and enhance mineral absorption, respectively [75]. Members of *Microbacteriaceae* produce 1-aminocyclopropane-1-carboxylate (ACC) deaminase, an enzyme that reduces ethylene levels, a plant stress hormone [78]. *Sphingomonadaceae* synthetize dehydrochlorinases, dehydrogenases, and halidohydrolases, enzymes that metabolize synthetic pesticides such as organochlorines [79,80]. Members of *Nocardioidaceae* and *Rhodobacteraceae* secrete phytohormones such as gibberellins and indole-3-acetic acid (IAA) that increase root length and improve nutrient and water absorption [81,82,83]. Taken together, these results suggest that tomato plant core microbiota could influence plant growth, disease resistance, and productivity. Nonetheless, additional studies should be performed to corroborate this notion. 

Interestingly, the present work and many other independent studies have identified *Burkholderiaceae*, *Caulobacteraceae*, *Chitinophagaceae*, *Flavobacteriaceae*, *Hyphomicrobiaceae*, *Rhizobiaceae*, *Rhodospirillaceae*, *Streptomycetaceae*, and *Xanthomonadaceae* as constitutive of the core microbiota in soil, substrate, and rhizosphere samples from tomato plants [18,63,84,85,86,87,88]. These results suggest that root tissues have evolved molecular mechanisms to recruit core microbiota populations [89]. Moreover, the co-occurrence of *Enterobacteriaceae* and *Oxalobacteraceae* in root and fruit tissues has been reported by other studies [26,30], supporting the idea that rhizosphere microbiota could influence the establishment of bacterial populations in the phyllosphere [90]. 

Additionally, in the present study, it was revealed that bacterial relative abundance and diversity were higher in substrates, followed by rhizosphere and fruit samples; comparable trends have been reported elsewhere [18,64,91,92]. Together, these results support the long-standing hypothesis [93] that suggests that stress generated by multiple and diverse environmental, biotic, and abiotic factors reduces the diversity and relative abundance of phyllosphere microbiota, when compared with rhizosphere microbiota [93,94].

### 4.2. Bacterial Relative Abundance Is Influenced by Conventional and Organic Soilless Culture Systems

It has been reported that tomato plants cultivated under organic SCSs have lower fruit yields, higher susceptibility to phytopathogens, as well as lower fruit firmness and weight [9,14,16,95]. It has been proposed that these deficiencies could be linked to dysbiosis in the tomato plant microbiota [20]. Herein, it was shown that conventional and organic plants have comparable bacterial abundance and richness in substrate, rhizosphere, and fruit samples; however, it was revealed that organic plants endured microbiota dysbiosis characterized by an increase in the relative abundance and dominance of some specific bacterial groups. Comparable phenomena have been reported in other studies with tomato, lettuce, and teak plants [67,96,97].

Specifically, the present study revealed that substrate, rhizosphere, and fruit samples under an organic SCS have altered relative abundance of 20, 21, and 12 families (listed in Figure 2e), respectively, with all of them members of Actinobacteria, Bacteroidetes, Firmicutes, and Proteobacteria phyla. Importantly, the dysbiosis characterized by a remarkable change in the relative abundance of *Enterobacteriaceae*, *Flavobacteriaceae*, *Hyphomicrobiaceae*, *Pseudomonadaceae Rhizobiaceae*, *Rhodobacteraceae*, and *Xanthomonadaceae* in substrate, rhizosphere, and fruit samples from the organic production system has been documented by many other studies [19,69,96,98,99,100], suggesting that these bacterial families could represent a microbial target that could be used to improve tomato plant health, productivity, and quality. 

To gain insights into the potential impact of these microbial populations on plant health and productivity, microbial biomarkers (LEfSe analysis) were identified. Particularly, it was revealed that most of the effects in the organic production system could be linked to *Flavobacteriaceae*, *Erythrobacteraceae*, *Bradyrhizobiaceae*, and *Nocardioidaceae* in substrates; *Caulobacteraceae*, *Chitinophagaceae*, *Flavobacteriaceae, Rhodobacteraceae*, *Streptomycetaceae*, in the rhizosphere; and *Enterobacteriaceae* in tomato fruit samples. These potential microbial biomarkers have been highlighted by other studies with tomato and lettuce cultivars [19,67,69,96,99]. 

Although additional and extensive studies are required to elucidate the effects of these bacterial biomarkers, numerous studies support the importance of these bacterial families on plant health and productivity. For instance, *Flavobacteriaceae* promotes phosphorus solubilization and pectin degradation; in environments under nutrient limitations, these bacterial groups could improve nutrient uptake and assimilation of organic compounds by plants [101,102]. *Bradyrhizobiaceae*, *Caulobacteraceae*, *Chitinophagaceae*, *Erythrobacteraceae*, *Nocardioidaceae*, and *Streptomycetaceae* promote biological nitrogen fixation by producing enzymes responsible for nitrogen assimilation [103,104,105,106,107,108] and increasing shoot and root biomass growth [109,110,111]. Thus, it could be possible that the reduction in the relative abundance of these bacterial biomarkers could be linked to the poor agricultural performance observed in organic SCSs [112].

Importantly, the present work revealed that *Enterobacteriaceae* is the most predominant member of tomato fruit microbiota; this finding has been documented in many other studies [26,29,66,113,114,115]. These results are of particular interest because genera within the *Enterobacteriaceae* family are important pathogens for tomato plants [116] and humans [117] as well as key members of the fruit spoilage microbiota [118,119]. The potential negative effects of *Enterobacteriaceae* in the organic production system could be related to the ability of this bacterial group to produce adhesins, phytotoxins, and proteases associated with plant pathogenesis [120,121]. Additionally, members of *Enterobacteriaceae* produce extracellular enzymes such as pectate lyases, polygalaturonases, pectin methylesterases, and pectin acetylesterases involved in fruit cell wall degradation [119]; an increased production of these enzymes has been linked to reductions in fruit firmness and shelf life [116,122]. 

Moreover, the high abundance of *Enterobacteriaceae* in tomato fruits is a major food safety problem. First, numerous studies have reported a high prevalence of potential enteropathogens such as *Enterobacter* spp. (range = 17–28%, [123,124,125]), *Escherichia coli* (range = 3–18%, [125,126,127]), *Klebsiella* spp. (range = 2–39%, [123,125]), and *Salmonella enterica* (range = 8–44%, [128,129]), all of them members of *Enterobacteriaceae*, in tomato fruits intended for human consumption. Second, in many countries, consumption of tainted tomatoes has been linked to *E. coli* [130], *S. enterica* [131,132,133,134,135,136], and *Shigella flexneri* [137] human outbreaks. For many years, tomato fruits have been described as *Enterobacteriaceae* pathogen carriers; however, in the last decade, numerous studies have shown that tomato fruits are an alternative host for colonization, replication, and propagation of *Enterobacteriaceae* [138,139,140]. Importantly, the present study revealed that a major effect of the organic SCS was a twenty-fold increment in the relative abundance of *Enterobacteriaceae* in tomato fruits. This finding was corroborated via an independent microbial molecular analysis with tomato fruits collected from a different harvest season. Together, these results suggest that the dysbiosis caused by the organic fertilization regime could potentially produce tomato fruits that are more susceptible to pathogen colonization; further studies are in progress to corroborate this idea. Furthermore, we hypothesize that the dysbiosis observed in the organic SCS could be attributed to the organic source of nitrogen, phosphorus, and potassium used in the present study. Numerous studies have shown that the use of organic fertilizers increases the relative abundance of different bacterial groups in soil and soilless cultivation systems [19,100,141,142,143,144]. Additional studies are in progress to evaluate this hypothesis.

## 5. Conclusions

In the present study, it was revealed that tomato plant microbiota was predominantly colonized by members of the Proteobacteria, Actinobacteria, and Firmicutes phyla families. Additionally, it was found that *Bacillaceae*, *Microbacteriaceae*, *Nocardioidaceae*, *Pseudomonadaceae*, *Rhodobacteraceae*, and *Sphingomonadaceae* colonized substrate, rhizosphere, and phyllosphere samples and are members of the tomato plant core microbiota.

Importantly, it was revealed that tomato plants cultivated under organic SCS endure a dysbiosis in substrates, roots, and fruits, characterized by an increased relative abundance of *Flavobacteriaceae*, *Rhodobacteraceae,* and *Enterobacteriaceae*; and a reduction of *Bradyrhizobiaceae*, *Caulobacteraceae*, *Chitinophagaceae*, *Erythrobacteraceae*, *Nocardioidaceae*, and *Streptomycetaceae*. Altogether, these results suggest that the dysbiosis observed in organic tomato plants could be responsible for the agricultural deficiencies reported for this production system. Importantly, the present study highlights a list of bacterial groups with biotechnological potential to promote plant health and production.

## Figures and Tables

**Table 1:** Sampling scheme for microbial population analyses of substrate, rhizosphere, and fruit samples from conventional and organic SCSs.

		Production Week ^1^			
		8	26	44	
Soilless Culture System	Niche	Composite Samples ^2^			Total of Samples
Conventional	Substrates	4	4	4	*n* = 12
	Rhizosphere	4	4	4	*n* = 12
	Fruits	4	4	4	*n* = 12
Organic	Substrates	4	4	4	*n* = 12
	Rhizosphere	4	4	4	*n* = 12
	Fruits	4	4	4	*n* = 12
		Total ^3^			*n* = 72

^1^ Weeks after seedling transplantation. ^2^ Individual collected samples were processed as composite samples (3 individual samples = 1 composite sample). ^3^ Total number of samples processed for *16S rRNA* gene-targeted sequencing.

## Data Availability

The authors declare that all relevant data supporting the findings of this study are included in this article.
